# Beyond Conventional: The New Horizon of Targeted Therapy for the Treatment of Advanced Non Small Cell Lung Cancer

**DOI:** 10.3389/fonc.2021.632256

**Published:** 2021-05-21

**Authors:** Alfredo Tartarone, Vittoria Lapadula, Concetta Di Micco, Gemma Rossi, Carlotta Ottanelli, Andrea Marini, Roberta Giorgione, Katia Ferrari, Martina Catalano, Luca Voltolini, Enrico Mini, Giandomenico Roviello

**Affiliations:** ^1^ Department of Onco-Hematology, Division of Medical Oncology IRCCS-CROB Referral Cancer Center of Basilicata, Rionero in Vulture, Italy; ^2^ Division of Medical Oncology, IRCCS Casa Sollievo della Sofferenza, San Giovanni Rotondo, Italy; ^3^ School of Human Health Sciences, University of Florence, Florence, Italy; ^4^ Respiratory Medicine, Careggi University Hospital, Florence, Italy; ^5^ Thoracic Surgery Unit, Careggi University Hospital, Florence, Italy; ^6^ Department of Health Sciences, Section of Clinical Pharmacology and Oncology, University of Florence, Florence, Italy

**Keywords:** targeted therapies, oncogenic drivers, genetic testing, non small cell lung cancer, MET

## Abstract

In the last few years the advent of targeted therapies against oncogenic drivers significantly improved the survival of non small cell lung cancer (NSCLC) patients with a favourable toxicity profile. Therefore, genetic testing, including at least EGFR mutations and ALK/ROS1 rearrangements, should be performed in all NSCLC patients (in particular with adenocarcinoma) who received a diagnosis of advanced disease. This review focuses on novel druggable oncogenic drivers, such as MET exon 14 mutations/MET amplification, RET fusions, BRAF V600E mutations, KRAS G12C mutations, NTRK rearrangements, and HER2 alterations.

## Introduction

The recently seen improvements in NSCLC outcomes are mainly related to the advent in clinical practice of immunotherapy in non-oncogene driven cancers and of targeted therapies in tumours with druggable oncogenes. Overall targeted therapies demonstrated not only to increase survival but also patient’s quality of life, due to their efficacy and favourable toxicity profile. In addition some of these drugs, such as osimertinib or alectinib, that are able to penetrate the blood-brain barrier, also showed a high effectiveness in treating or preventing brain metastases, a common clinical problem in NSCLC patients (1, 2).

The majority of oncogene-addicted NSCLC are adenocarcinomas, reason for which guidelines suggest that all patients with advanced adenocarcinoma should be tested for oncogenic drivers (3–5). In particular, testing for EGFR mutations and ALK or ROS1 rearrangements are now considered mandatory, while testing for emerging targets such as BRAF V600E mutations, MET exon 14 mutations, RET fusions, HER2 and NTRK1 are suggested. Anyhow several oncology services also routinely test for BRAF V600E mutations in view of the recent approval of BRAF/MEK inhibitors for metastatic BRAF V600E–mutated NSCLC patients.

However, even though the main guidelines recommend performing genetic testing to find out molecular disease drivers, a recent survey conducted by the International Association for the Study of Lung Cancer (IASLC) showed that 33% of respondents were unaware of the most recent guidelines for molecular testing and that less than half of the patients in their country receive molecular testing (6). As reported by the same survey, the main barriers to testing were cost, quality (inadequate tissue quality, lack of technical expertise in the laboratory, etc.), turnaround time and lack of awareness.

A multicenter Italian observational study of biomarker screening in daily clinical practice conducted from May 2017 to October 2017 in 13 institutions (N=1612 patients) reported that only 50.8% requests were related to driver mutations with target agents already available at the preplanned time, while 49.2% were associated with PD-L1, ROS1, KRAS and others (7). All participating centers considered multiplex genotyping assays such as next generation sequencing (NGS) as first approach.

**Figure 1:**
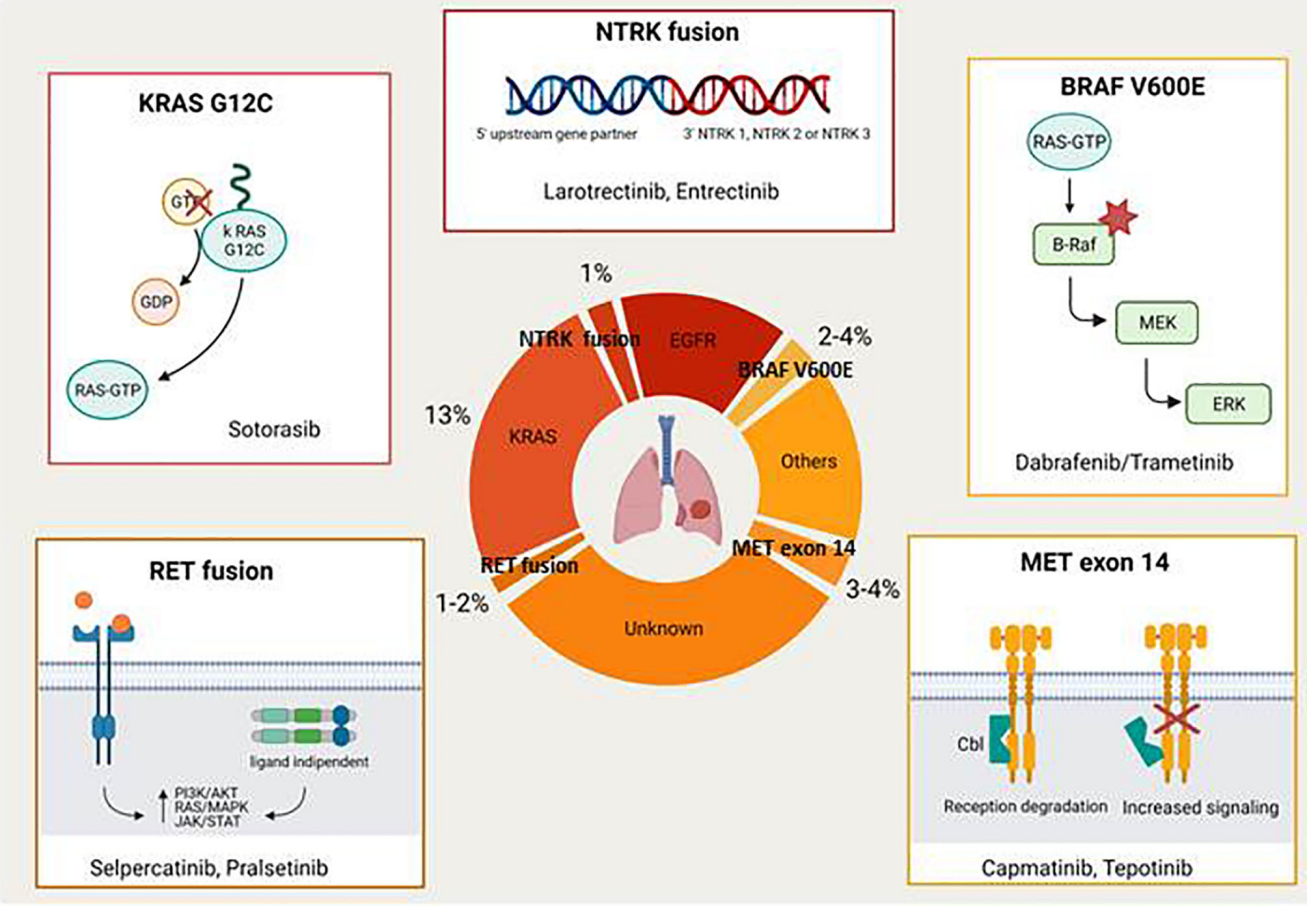
Novel druggable oncogenic drivers in NSCLC.

The majority of driver mutations are targetable by the EGFR tyrosine kinase inhibitors (TKIs) (e.g. gefitinib, erlotinib, afatinib, and osimertinib in EGFR-mutant NSCLC; crizotinib, ceritinib, brigatinib, lorlatinib, and alectinib in ALK rearranged tumours; crizotinib in ROS1 rearranged disease). This review focuses on novel druggable oncogenic drivers such as MET Exon 14 mutations/MET amplification, RET fusions, BRAF V600E mutations, KRAS G12C mutations, NTRK rearrangements, and HER2 alterations (**Figure 1**) .

## Met Exon 14 Mutation/Met Amplification

The mesenchymal-epithelial transition (MET) proto-oncogene located on chromosome 7 encodes a receptor tyrosine kinase with an extracellular alfa-subunit and a transmembrane beta-subunit linked by a disulfide bond. MET oncogene drives specific pathways through activation by its ligand hepatocyte growth factor (HGF) and participates in mechanisms of epithelial to mesenchymal transition. When MET exon 14 is not transcribed, there is decreased degradation of the MET receptor tyrosine kinase with a corresponding increase in signalling. It is known that MET and its ligand play a role during embryogenesis, are essential for organ protection/regeneration and also contribute to the regulation of the immune system. Aberrant regulation of the MET/HGF axis with subsequent alteration of downstream signalling pathways has been associated with tumourigenesis, invasiveness and cancer progression (8, 9). MET aberrations include overexpression, gene amplification and MET exon 14 skipping mutations. MET amplification represents also the most common resistance mechanism to osimertinib, accounting for about 15% of the cases (10). This has provided the rationale for various clinical trials exploring the combination of MET and EGFR TKIs in patients with mutant EGFR and MET amplified NSCLC after progression on EGFR TKIs (11). Cases of NSCLC patients with MET exon 14 mutations or MET amplification who responded to crizotinib or other MET inhibitors were already reported in literature several years ago (12, 13).

Clinical characteristics associated with MET exon 14 mutations were reported in **Table 1A**.

**Table 1:** Clinical characteristics associated with MET exon 14 mutation/MET amplification **(A)** and RET deregulation **(B)**.

A	B
Older age (≥ 70 years)	Younger age (≤ 60 years)
Affects both smokers and non-smokers	Never-smoker status
Detectable in 3-4% of NSCLC patients	Mutually exclusive with other mutations
Can occur in any subtype of NSCLC (high frequency in pulmonary sarcomatoid carcinoma)	Observed in 1-2% of NSCLC (adenocarcinoma) patients as well as in other cancer (in particular thyroid cancer)
No gender specificity	Signet ring cells in ≥ 10% of tumor cells
	Poorly differentiated tumors
	Early lymph-node metastases
	Frequent presence of brain metastases
	Low response rate with chemotherapy

Recently, two large studies published in the NEJM demonstrated the activity of the anti MET capmatinib and tepotinib in this patient population (14, 15). The GEOMETRY mono-1 study investigated the activity of capmatinib, an orally bioavailable inhibitor of the MET receptor, in MET-positive NSCLC patients. In this study patients were assigned to cohorts on the basis of MET status (MET exon 14 skipping mutation or MET amplification) and previous therapies (14). A total of 364 patients were included in the various cohorts; among patients with NSCLC with a MET exon 14 skipping mutation the overall response rate (ORR) was 41% in 69 pretreated patients and 68% in 28 patients who had not received treatment previously, while the median duration of response was 9.7 and 12.6 months, respectively. Limited efficacy was recorded only in previously treated patients with MET amplification who had a gene copy number of less than 10 (ORR in 7–12% of patients). Among patients with MET amplification and a gene copy number ≥ 10 ORR was 29% in pretreated patients and 40% in chemotherapy-naive, in any case lower RRs than those reported in patients with MET exon 14 skipping mutation.

Interestingly, capmatinib demonstrated activity also in patients with brain metastases (with responses observed in 12 out of 13 patients). About toxicity, the most frequently reported adverse events (AEs) were peripheral oedema (51%) and nausea (45%).

The VISION study evaluated the clinical activity of tepotinib, another oral MET inhibitor, in chemotherapy-naïve or pretreated patients (> 2 courses of previous therapy) with a confirmed MET exon 14 skipping mutation (cohort A) or with MET-amplified disease (cohort B); currently cohort C is enrolling patients with MET exon 14 skipping mutations for confirmatory analysis of the results reported in cohort A (15). Testing of MET exon 14 skipping mutations was performed analysing circulating free DNA (cfDNA) obtained from plasma or RNA extracted from fresh or archivial tumour tissue. Recently the authors reported the results for cohort A, which has completed recruitment. Among the 99 evaluable patients the ORR was 46% (48% in the liquid biopsy group and 50% in the tissue biopsy group), with a median duration of response of 11.1 months. The response rates were similar regardless of the number of previous therapies. About toxicity grade ≥ 3 AEs were reported in 28% of the patients, including peripheral oedema in 7% that represented the main toxic effect.

The U.S. Food and Drug Administration (FDA) granted accelerated approval to capmatinib and tepotinib, on May 2020 and February 2021 respectively, for patients with metastatic NSCLC harboring MET exon 14 skipping alterations.

The findings from the GEOMETRY and VISION study indicate not only that MET exon 14 skipping mutation represents a new therapeutic target but also the importance of routine testing for these alterations.

## RET Fusions

The RET proto-oncogene encodes a transmembrane receptor tyrosine kinase with an extracellular, a transmembrane and an intracellular tyrosine kinase domain (16). RET receptor, that binds growth factors of the glial derived neurotropic factor family, when is deregulated becomes a potent oncoprotein (17). The most common identified RET aberrations are mutations, fusions and amplifications. RET germline mutations are associated with multiple endocrine neoplasia type 2A (MEN2A), MEN2B and familial medullary thyroid carcinoma.

Main clinical characteristics associated with RET deregulation are listed in **Table 1B**. Recently, were reported data regarding the use of two RET inhibitors, selpercatinib and pralsetinib, in patients with advanced RET fusion positive NSCLC (18, 19). The phase 1 to 2 LIBRETTO 001 study, conducted in RET fusion positive NSCLC patients either previously treated with platinum-based chemotherapy or chemotherapy-naïve, showed encouraging results for selpercatinib, a novel inhibitor of RET kinase (18). The authors reported an ORR of 64% in 105 pretreated patients with a median duration of response of 17.5 months; among 39 chemotherapy-naïve patients the ORR was 85% and 90% of the responses were ongoing at 6 months. Notably, 10 out of 11 patients (91%) with measurable central nervous system (CNS) metastases at enrolment obtained an objective intracranial response, including 3 complete responses. The most common reported AEs of grade ≥ 3 were hypertension (14%), hypertransaminasemia (12%) and hyponatremia (6%); however, only 2% of patients discontinued selpercatinib due to a drug related AE.

Phase I/II ARROW study evaluated the activity of another RET inhibitor, pralsetinib (BLU-667), in RET+ solid cancers, including NSCLC (ClinicalTrials.gov Identifier: NCT03037385). In the dose-escalation phase of the study the recommended dose has been determined at 400 mg once daily. Updated analysis of this study regarding 116 NSCLC patients showed an ORR of 65% (61% in patients with prior platinum treatment, 73% in patients with no prior systemic therapy), including 6% of complete responses (19). Most treatment related AEs were grade 1 to 2 and included hypertransaminasemia, constipation, hypertension and anemia. Similarly to selpercatinib, pralsetinib demonstrated an high activity against CNS metastases. AcceleRET Lung, an ongoing phase III study, will evaluate the efficacy and safety of pralsetinib versus standard of care for first-line treatment of advanced/metastatic RET fusion-positive NSCLC (ClinicalTrials.gov Identifier: NCT04222972).

Updated National Comprehensive Cancer Network Clinical Practice Guidelines in Oncology (NCCN Guidelines^®^) for NSCLC indicate selpercatinib or pralsetinib as a preferred treatment option for patients with RET fusion-positive NSCLC as a first-line or subsequent therapy (20).

## BRAF V600E Mutations

BRAF mutations are identified in 2% to 4% of NSCLC and BRAF V600E point mutations account for 50% of these cases. BRAF mutations determine an activation of the mitogen-activated-protein-kinase (MAPK) pathway that regulates cellular growth. In addition, BRAF mutations represent an emerging mechanism of resistance to EGFR-TKIs that has been reported in 1% to 2% of cases (21).

Planchard et al. enrolled patients with advanced BRAF V600E mutant NSCLC in three cohorts (22–24). In the cohort A untreated or previously treated patients received dabrafenib (150 mg twice daily) as monotherapy, in the cohort B previously treated patients received the combination of (dabrafenib 150 mg twice daily) and trametinib (2 mg once daily), in the cohort C untreated patients were treated with dabrafenib (dabrafenib 150 mg twice daily) plus trametinib (2 mg once daily). The first two cohorts showed a higher efficacy of the combination than the one observed with dabrafenib alone (ORR 67% *vs* 33%), still bearing in mind the limits of an indirect comparison. Results of the cohort C, including untreated patients with who have received dabrafenib 150 mg twice daily and trametinib 2 mg daily, confirmed the efficacy of this combination therapy with an ORR of 64% and a median progression-free survival (PFS) of 10.9 months; the toxicity of the combination was manageable, being the most serious AEs pyrexia (11%), aspartate aminotransferase increase (8%) and ejection fraction decrease (8%). Main guidelines recommend dabrafenib plus trametinib for metastatic BRAFV600–mutated NSCLC patients (3, 20).

Vemurafenib, another BRAF inhibitor, was administered in 115 pretreated NSCLC patients (100 with BRAFV600 mutations and 15 with BRAF.nonV600 mutations) as part of the AcSè program conducted by the French National Cancer Institute (25). This study demonstrated the activity of vemurafenib in BRAFV600 mutated patients (ORR 44.9%, median PFS 5.2 and median OS 10 months, respectively), but not in patients with other BRAF mutations. The safety profile was comparable with that usually observed with dabrafenib.

## KRAS G12C Mutations

Carcinogenic Kirstein Rat Sarcoma viral oncogene homolog (KRAS) mutation is the most common mutation in NSCLC, accounting for approximately 30% of adenocarcinomas and 5% of squamous lung cancers. KRAS encodes small G proteins which are involved in several pathways such as proliferation, differentiation and apoptosis. KRAS mutations, that mainly occur in codon 12 (13% of NSCLC), 13 and 61, determine a loss of intrinsic GTPase activity with subsequent effects on cell proliferation signals and tumourigenesis (26). Up until now it has been very difficult to target K-RAS, probably due to its ability to activate multiple mechanisms of escape under the selective pressure of the treatment (27).

However, recent results from the phase 1 CodeBreak 100 trial (ClinicalTrials.gov Identifier: NCT03600883) with sotorasib (AMG 510), a first in class inhibitor of the KRAS G12C mutation, showed encouraging activity in heavily pre-treated advanced NSCLC and other solid tumours harbouring the KRAS G12C mutation (28). The study that included a total of 129 patients (59 with NSCLC, 42 with colorectal cancer, 28 with other tumours) showed an ORR of 32.2%, 88% of disease control (objective response or stable disease) and a median PFS of 6.3 months among patients with NSCLC. The most common reported AEs were diarrhea (any grade 29.5%, grade ≥ 3 3.9%), fatigue (any grade 23.3%, grade ≥ 3 2.3%) and nausea (any grade 20.9%, grade ≥ 3 1.6%). As reported by LoRusso and Sebolt-Leopold in their editorial on the NEJM, this trial represents the first step in “drugging the undruggable” (29).

All over the world more than 100 studies are currently ongoing to evaluate the role of novel agents administered alone or in combination in KRAS mutant NSCLC patients (30).

## NTRK Fusions

Neurotrophic receptor tyrosine kinase (NTRK) gene fusions, that encode the tropomyosin receptor kinase (TRK) proteins, are genomic alterations that can act as an oncogenic driver, promoting cell proliferation and survival in tumour cell lines (31). NTRK fusions have been found across multiple tumour types from both adult and paediatric patients. Their frequency varies from <5% in cancer types including lung, pancreatic, colorectal, melanoma, breast cancers and other solid or haematological cancers, up to 25% in tumours including gastrointestinal stromal tumours and thyroid cancer, to >90% in rare tumours types such as infantile fibrosarcoma, cellular or mixed congenital mesoblastic nephroma and mammary analogue secretory carcinoma (MASC). Among the various TRK inhibitors that have been investigated in the last few years larotrectinib and entrectinib are the ones with the most promising development (32–34). Larotrectinib was granted approval by U.S. Food and Drug Administration (FDA) and European Medicines Agency (EMA), in 2018 and 2019 respectively, for the treatment of patients with advanced NTRK fusion positive solid tumours based on the results of three distinct single-arm trials including also NSCLC patients (35). In particular, larotrectinib demonstrated in the first 55 enrolled patients an ORR of 75% (including 13% of complete responses) with 55% of the patients remained progression-free at 1 year. In the updated pooled efficacy analysis that included a total of 159 patients (153 evaluable for response) 121 patients (79%) had an objective response (16% complete response) with a median PFS of 28.3 months (36). Few serious AEs were observed in these trials, considering that grade 3 to 4 AEs were reported in 13% and <1% of patients respectively. The most common reported grade 3 to 4 AEs were increased alanine aminotransferase, anemia, neutropenia, fatigue and pyrexia.

Recently, also entrectinib received FDA and EMA approval for the same patient population. The decision was based on the results of three small phase 1 to 2 trials (STARTRK-1, STRTRK-2, ALKA) including 54 patients with advanced NTRK+tumours (37). In these studies the authors reported an ORR of 59.3%, including 7% complete responses, with a median duration of response of 12.9 months. In particular, in the cohort of patients with NSCLC (n=10) the ORR was 70.0%; moreover, among NSCLC patients with baseline CNS disease (n=6) 4/6 had an intracranial response (2 complete, 2 partial), 1 stable disease while 1 was not evaluable (38). Entrectinib was well tolerated with AEs of grade 1 to 2 being the most observed and a discontinuation rate of 4.4%. Entrectinib has also received FDA and EMA approval for the treatment of ROS1 positive metastatic NSCLC in view of the positive results obtained in this setting of patients in the STARTRK-1, STARTRK-2, ALKA-372-001, and STARTRK-NG trials. Finally, preliminary results showed the efficacy of selitrectinib (LOXO-195), a next generation TRK inhibitor, in TRK fusion-positive patients with resistance to prior anti-TRK kinase therapy (ClinicalTrials.gov Identifier: NCT03215511) (39).

In the era of precision medicine larotrectinib and entrectinib represent one of the few examples of the so-called agnostic therapies; in fact, they target specific genomic anomalies regardless of tumour site of origin.

## HER2

The human epidermal growth factor receptor 2 (HER2) is a member of the epidermal growth factor receptor family having tyrosine kinase activity. Similarly to MET three principal HER2 alterations can be identified: HER2 amplification, HER2 overexpression and HER2 mutations. HER2 has a key role in signal transduction and oncogenesis; in particular, HER2 aberrations, including both amplification and mutations, have been considered as oncogenic drivers that contribute to 2% to 6% of lung adenocarcinoma. In addition, as well as MET amplification, also HER2 amplification represents an important mechanism for acquired resistance to EGFR TKIs (40). So far several clinical trials conducted in NSCLC patients with HER2 aberration showed a modest efficacy of small molecule TKIs (e.g. afatinib, dacomitinib), anti-HER2 antibodies administered alone (e.g. trastuzumab) or in association with chemotherapy (e.g. trastuzumab+carboplatin/paclitaxel) or antibody-drug coniugate (ADC) (e.g.T-DM1) (41). However, at the 2020 ASCO Meeting preliminary results of the phase 2 DESTINY Lung-01 trial (ClinicalTrials.gov Identifier: NCT03505710) demonstrated encouraging activity of trastuzumab deruxtecan treatment, a novel ADC, in patients (n=42) with relapsed/refractory HER2-mutant NSCLC enrolled in the cohort 2 of the study. In fact, the authors reported an ORR of 61.9% and a median PFS >1 year with a median follow-up of 8 months (42).

On May 2020, the FDA granted breakthrough therapy designation to trastuzumab deruxtecan for the treatment of patients with metastatic and pretreated NSCLC whose tumours have a HER2 mutation.

At the 2020 World Conference on Lung Cancer (WCLC) were presented preliminary results of the patients with relapsed/refractory HER2-overexpressing (IHC 3+ or 2+) NSCLC enrolled in the cohort 1 of the same study (43). Patients with HER2 overexpression achieved an ORR of 24.5% and a disease control rate (DCR) of 69.4% with a median PFS of 5.4 months. Drug-related AEs were grade ≥ 3 in 55.1% of the patients and the most common AE was neutropenia. Drug-related interstitial lung disease (ILD) was observed in 8 patients.

## Conclusion

Over the last years NSCLC has become an example of how precision medicine can significantly improve patient outcomes. In fact in advanced NSCLC patients harboring driver mutations and treated with targeted therapies we achieved results that traditional chemotherapy never gave us.

As previously reported, novel targeted agents such as capmatinib/tepotinib, selpercatinib/pralsetinib, dabrafenib+trametinib, sotorasib, larotrectinib/entrectinib, trastuzumab-deruxtecan are already or will be soon available for special subsets of patients with metastatic NSCLC.

In addition recent data of the phase III ADAURA trial, that evaluated the efficacy of osimertinib in EGFR mutated patients in the adjuvant setting, suggest that targeted therapy will play a role also in NSCLC patients with an oncogene addicted early disease (44).

In conclusion, identifying novel molecular subsets, developing much more efficient targeted therapies as well as performing genomic testing in clinical practice, as recommended by main guidelines, represents the next challenge and the best way to achieve the goal of giving the right drug to the right patient.

## Author Contributions

Conceptualization, GiR and AT. Writing—original draft preparation, AT, GeR, LV, KF, CO, GiR, RG, CM, and VL. Writing—review and editing, EM. Supervision, EM. All authors contributed to the article and approved the submitted version.

## Conflict of Interest

The authors declare that the research was conducted in the absence of any commercial or financial relationships that could be construed as a potential conflict of interest.
